# Voluntary initiation of movement: multifunctional integration of subjective agency

**DOI:** 10.3389/fpsyg.2015.00688

**Published:** 2015-05-22

**Authors:** Patrick Grüneberg, Hideki Kadone, Kenji Suzuki

**Affiliations:** ^1^School of Global Japanese Studies, Meiji UniversityTokyo, Japan; ^2^Artificial Intelligence Laboratory, University of TsukubaTsukuba, Japan; ^3^Center for Innovative Medicine and Engineering, University of Tsukuba HospitalTsukuba, Japan; ^4^Center for Cybernics Research, University of TsukubaTsukuba, Japan

**Keywords:** subjectivity, agency (psychology), motion, multimodality, multifunctionality, neurorehabilitation, assistive robotics

## Abstract

This paper investigates subjective agency (SA) as a special type of efficacious action consciousness. Our central claims are, firstly, that SA is a conscious act of voluntarily initiating bodily motion. Secondly, we argue that SA is a case of multifunctional integration of behavioral functions being analogous to multisensory integration of sensory modalities. This is based on new perspectives on the initiation of action opened up by recent advancements in robot assisted neuro-rehabilitation which depends on the active participation of the patient and yields experimental evidence that there is SA in terms of a conscious act of voluntarily initiating bodily motion (phenomenal performance). Conventionally, action consciousness has been considered as a sense of agency (SoA). According to this view, the conscious subject merely echoes motor performance and does not cause bodily motion. Depending on sensory input, SoA is implemented by means of unifunctional integration (binding) and inevitably results in non-efficacious action consciousness. In contrast, SA comes as a phenomenal performance which causes motion and builds on multifunctional integration. Therefore, the common conception of the brain should be shifted toward multifunctional integration in order to allow for efficacious action consciousness. For this purpose, we suggest the heterarchic principle of asymmetric reciprocity and neural operators underlying SA. The general idea is that multifunctional integration allows conscious acts to be simultaneously implemented with motor behavior so that the resulting behavior (SA) comes as efficacious action consciousness. Regarding the neural implementation, multifunctional integration rather relies on operators than on modular functions. A robotic case study and possible experimental setups with testable hypotheses building on SA are presented.

## 1. Introduction

While unimodal and multimodal integration are usually concerned with the processing of sensory input and perceptual consciousness, an analogous point for shifting from a unimodal to a multimodal setup can be made in the case of action consciousness. As action consciousness mainly builds on behavioral functions of action initiation and control, the uni-/multimodal-distinction turns into the distinction between *unifunctional* and *multifunctional integration* of behavioral functions. Unifunctional integration (binding) is mainly applied in order to explain the phenomenal experience of action consciousness in terms of a sense of agency (SoA), authorship, or control (Gallagher, 2000, Gallagher, 2012)^1^. According to SoA as an experiential concept, current phenomenology of action as well as psychological and neurocognitive research on action do not leave any space for bodily motion being initiated by the conscious agent. Even if voluntary initiation is well-known in neuroscientific research on motion, the question “Does Consciousness Cause Behavior?” (Pockett et al., 2006) tends to be negatively answered. While initiation is usually left to the locomotor system, the conscious agent is limited to experiencing action *post-hoc*. Therefore, the conscious subject merely echoes motor performance (Haggard and Johnson, 2003) and is not regarded to be an efficacious agent who causes bodily motion (Bayne and Levy, 2009). In this view, action consciousness is an epiphenomenal addition to sub-personal processes of the locomotor system.

The concept of multisensory (multimodal) integration emerged as an alternative approach to the problem of sensory integration, i.e., how different sensory modalities interact in order to form coherent representations of objects or processes underlying sensory input. According to the standard view, sensory modalities are processed independently in their respective brain areas and later on integrated by means of binding. As this kind of integration depends on single modalities the standard approach can be referred to as *unimodal integration* or just *binding*. By postulating multisensory neurons, this standard view has been challenged (Calvert and Thesen, 2004, Alais et al., 2010). Multimodality implies that there is no one-to-one mapping of sensory input to a certain brain area. Instead, different sensory modalities can be processed by one and the same area so that integration already takes place at the primary level of sensory processing. This new perspective, referred to as *multimodal (multisensory) integration*, contains farreaching implications for the functional organization of the brain as well as for the cognitive and phenomenal (conscious) aspects of sensory processing and action control (Musseler et al., 2014).

Opposed to this common view, robot-assisted rehabilitation (Tejima, 2001, Feil-Seifer and Mataric, 2005) opens up a new perspective on the phenomenology of action. The rehabilitative application of robotic devices which crucially depend on the active participation of the patient (Hogan et al., 2006, Duschau-Wicke et al., 2010), yields experimental evidence that there is action consciousness prior to conducted motion. There is subjective agency (SA) in terms of a conscious and therefore subjective act of voluntarily initiating bodily motion (Zhu, 2004, Kawamoto et al., 2010). “Subjective” concerns the individual conception of reality. An agent is subjective if her behavior is not completely predefined in terms of its task-orientation and (functionally defined) course of action (Grüneberg and Suzuki, 2014). In this view, action consciousness is a particular instance of subjective in terms of autonomous behavior. Accordingly, “voluntary” here means that the human agent initiated the motion of her body based on her (spontaneous) decision and regardless whether there has been a previous external stimulus provoking a reflex or any internal constraint like Libet's urge. It is also irrelevant whether motion actually occurs as SA's efficacy concerns the release of a controlling neural signal (motor program) which may or may not result in bodily motion.

Conceding that robotic research serves as a source for investigating human cognition and behavior (Oudeyer, 2010, Morse et al., 2011), we use robotic experiments for identifying SA^2^. While action in general is a long-known candidate for integrating different modalities (Gallese, 2000), SA—compared to SoA—suggests a basically different type of action consciousness which in turn asks for a different explanation. In the same way as the multimodal approach suggests intersensory integration at the basic neuronal level of sensory processing, we suggest that *interfunctional* integration already occurs at the basic neuronal level of action initiation. Accordingly, our focus lies on the *functional organization* of action consciousness which allows SA as efficacious action consciousness. Following this approach, this paper aims at revealing a substantial constituent of action consciousness and at suggesting an explanation for SA as a case of multifunctional integration.

The remainder of this paper is divided into two parts. Section 2 and 3 identify SA as a distinct type of efficacious action consciousness. Section 4 and 5 investigate SA as a case of multifunctional integration and present experimental evidence as well as hypotheses based on SA. Because SA as an efficacious capacity is usually not regarded as a feature of action consciousness, we will first identify SA. For this purpose, we introduce robotic neurorehabilitation and in particular focus on the patient's role in the therapeutic process (Section 2.1). By means of analyzing the implementation and effects of robotic neurorehabilitation, we argue that SA is efficacious in terms of voluntary initiation of motor programs (Section 2.2). Then we show that SA does not fall under common action consciousness (SoA). Due to the experiential stance of SoA (Section 3.1) and the corresponding functional organization (unifunctional integration), SoA does not capture SA as a conscious and at the same time efficacious capacity (Section 3.2). The identification of SA and its exclusion from common action consciousness lead to the conclusion that SA comes as a distinct type of the phenomenology of action and is classified as a phenomenal performance (Section 3.3). Based on this finding, we argue that the brain must be able to implement SA (Section 4.1) and present a functional organization (multifunctional integration) which allows for SA as efficacious action consciousness (Section 4.2). Finally, we illustrate SA by means of a case study of a robotic device for lower limb rehabilitation (Section 5.1). Hypotheses regarding neurorehabilitation and athletic sport are suggested which promise to gain insight into the link between SA and its implementation in motor behavior together with the detection of effects of neurorehabilitation (Section 5.2).

## 2. Subjective agency in the course of robotic neurorehabilitation

In recent years, exoskeleton robots have been developed for the rehabilitation of impairments of upper and lower limbs. Traditional physiotherapy follows a bottom-up approach in terms of acting on the (distal) physical level in order to influence the neural system. In comparison, robotic devices build on therapeutic top-down control for the purpose of neurorehabilitation (Belda-Lois et al., 2011). Hereby, neurorehabilitation depends on the state of the brain after a stroke or other damage and not on the physical level of the impaired limbs. In order to exploit neuroplasticity for rehabilitative purposes and motor learning, the patient's voluntary involvement in the therapeutic process is essential (Hogan et al., 2006) similar to cognitive-behavioral therapy where therapeutic effects also depend on the conscious modification of thought or behavior by the patient herself (Brewin, 1996; McKay et al., 2015). Therefore, robotic rehabilitation devices for upper (Maciejasz et al., 2014 for an overview) and lower limbs (Daz et al., 2011 for an overview) enable the patient to move her impaired limbs voluntarily despite the impairment.

### 2.1. From being moved to voluntary initiation: device control by biosignals

The standard electromechanical approach to exoskeleton robots consists mainly of replacing motion support delivered by a physiotherapist. It is the task of an exoskeleton robot to move a limb according to a predetermined kinematic trajectory; thereby the patient makes use of the autonomous motion generated by the robot (Belda-Lois et al., 2011). *Thus, the patient is being moved*. Accordingly, purely mechanically based exoskeleton robots do not fully utilize a top-down approach by making use of remaining brain capacities and increasing the patient's involvement in motion generation because they do not consider the patient's intention to move her limbs voluntarily. Motion support remains passive as a purely mechanical and automated process closer to common physiotherapeutic support.

In order to increase the patient's participation, the control strategy of the robotic device has to be extended for implementing active support. Instead of letting the robot execute predetermined kinematic patterns, biosignals of patients can be exploited for the control of the robotic device (de Almeida Ribeiro et al., 2013). As especially EMG signals of neural muscle activity can be detected even in patients with severe impairments, these signals can be used to interpret the patient's intention to move, i.e., for human intention estimation (Suzuki et al., 2007). Thus, the patient is no longer being passively moved by the robotic device, but is enabled to control the robot directly by her capacity to voluntarily initiate bodily motion. Motion support is delivered according to the patient's needs^3^.

### 2.2. Closing the proprioceptive loop

**Figure 1:**
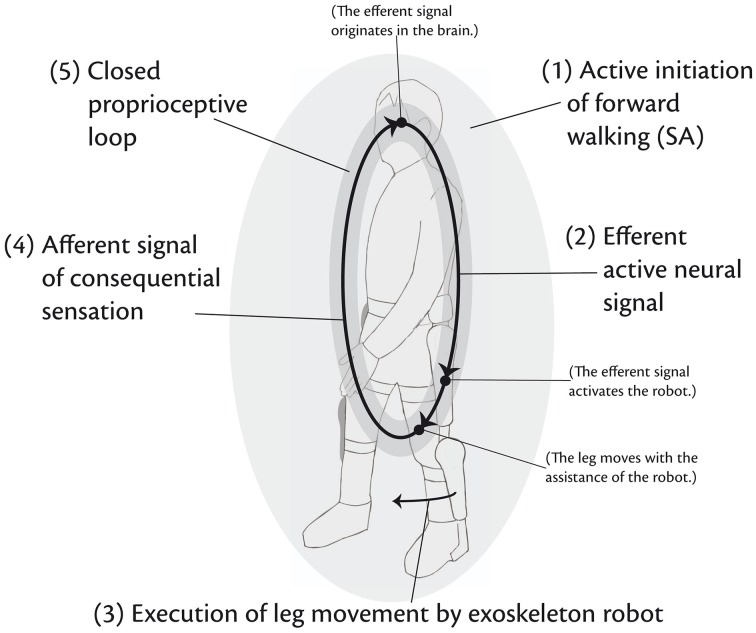
**SA initiating the proprioceptive loop**. Based on “SA of forward walking” (1), an efferent active neural signal of the intended motion is released (2). The robotic device (in this example a lower-limb exoskeleton robot) detects the signal and supports the execution of leg movement (3) so that an afferent signal of consequential sensation goes back to the brain and signals that a motion has been executed successfully despite of the impairment (4). The closed proprioceptive loop of physical interaction (5) is supposed to enhance neurorehabilitation of the brain. Contrary to locating SA in the brain, conscious acts are regarded here as acts of the entire agent comprising the central nervous system as well as the actuators.

The obvious reason for arguing for SA lies in the therapeutic effects achieved by devices using biosignals (Kawamoto et al., 2013, Maciejasz et al., 2014). Lacking a decisive neuroscientific explanation for these effects, the following hypothesis might serve as a starting point to understand the implementation and effects of robotic neurorehabilitation: *Depending on SA, robotic devices allow for the closing of the proprioceptive loop* (Kawamoto et al., 2013) *of physical interaction between the efferent active neural signal and the afferent signal of consequential sensation of the intended motion and thereby enhance neurorehabilitation in that the brain detects successful initiation and execution of motion despite of the impairment*. According to this hypothesis, the therapeutic effects of a recovery of motivity and the underlying recovery of the corresponding brain regions are derived as follows (cf. Figure 1; for the sake of simplicity, we will illustrate the hypothesis by lower limb rehabilitation of forward gait which could be replaced by any other limb):

**Table:** 

0	In case of locomotively impaired patients, there is no automatic (sub-personal) initiation of forward walking as the neural signals are not sufficient in order to activate the leg muscles. The patient remains in a resting position when no therapeutic actions are taken.
1	After being equipped with an exoskeleton robot, the patient voluntarily initiates forward walking (SA), i.e., consciously issues the command to move.
2	The neural activity of motor commands issued by the patient can be detected in the leg muscles: there is an efferent active neural signal.
3	The exoskeleton detects this signal by its EMG sensors and launches its motion support: actual walking motion is executed.
4	Due to the execution of a walking motion, an afferent signal of consequential sensation goes back to the brain and signals that a motion has been executed successfully.
5	The proprioceptive loop is closed. The brain regions responsible for motion control can chalk up a successful motion and remain active or become (partially) restored^4^.

The key assumption in this hypothetical sequence of the therapeutic process concerns the recovery of the brain regions responsible for motion control by means of closing the proprioceptive loop of efferent and afferent neural signals. Even if current research does not yield a final neuroscientific explanation for this effect, two findings are nevertheless obvious: Following (0), there is no automatic (sub-personal) initiation of motion in the therapeutic setting. This means that the sub-personal mechanisms of the locomotor system, which are usually held responsible for motion initiation (Haggard, 2005, Frith, 2013), no longer provide sufficient resources to initiate bodily motion automatically. The result is the obvious impairment of the patient and her corresponding inability to move her body. *Thus, firstly, in the case of patients with locomotive impairment there is no sub-personal (automatic) initiation of motion as the brain and/or the spinal cord are impaired to the extent that control and initiation of motion are no longer available automatically*.

Limited knowledge about neurorehabilitation does not challenge the clinical evidence (cf. Section 5.1) that there are significant therapeutic effects by robotic rehabilitation. So following (1) and (2), robotic therapy shows that there is a certain conscious and efficacious capacity of SA in order to voluntarily initiate motion. This finding can be directly concluded from the fact that the rehabilitation robot is only operated if the patient voluntarily (consciously) seeks to walk forward (Hogan et al., 2006, Eitam et al., 2013)^5^. If the patient does not voluntarily engage in the therapeutic process, nothing will happen (as stated above) and the patient's condition might even deteriorate. Accordingly, the *initiation* of the proprioceptive loop by means of the efferent active neural signal depends on the voluntary initiation by the patient. Regarding the motor-related objective of the initiation, behavioral research suggests that cognitive action control concerns the synergetic level of bodily motion (Latash et al., 2007). Voluntary motor programs identified by Ivanenko et al. are possible candidates to implement initiated motion physically (Ivanenko et al., 2004, Lacquaniti et al., 2012). They suggest five basic locomotion motor programs for gait which are possibly superimposed by voluntary motor programs depending on the subject's control^6^. These motor programs are released even if no actual motion occurs. *Thus, secondly, SA is efficacious in terms of voluntary initiation of motor programs*.

## 3. Unifunctional integration and the sense of agency

In general, conscious experience is supposed to form a particular and rather problematic case of unimodal integration as the coherence of objects and the phenomenal homogeneity of experience ask for a relatively high degree of integration. The same counts for motor behavior and is here referred to as *unifunctional integration*: Basic functions of motor behavior are integrated (bound) in order to make certain aspects of motor behavior contents of phenomenal experience. Action consciousness is usually spelled out in terms of SoA which forms a result of unifunctional integration. After a brief look at the common phenomenology of action and the underlying experiential stance toward agency (Section 3.1), we will turn to Pacherie (2008) as she links a strong phenomenology of action with its neural implementation. In particular, we examine how Pacherie draws on binding in order to explain SoA. It can be shown by means of the functional organization of SoA that unifunctional approaches to action consciousness such as SoA inevitably lead to *post-hoc* (experiential) action consciousness and therefore do not capture SA as an efficacious capacity (Section 3.2). Finally, the identification of SA (cf. Section 2) and its exclusion from common action consciousness (Section 3.2) lead to the conclusion that SA comes as a distinct type of the phenomenology of action which will be classified as a phenomenal performance (Section 3.3).

### 3.1. Senses and experiences: the experiential stance toward agency

The phenomenology of action is usually regarded as thin and evasive to the extent that its phenomenal content cannot be identified clearly (Metzinger, 2006). However, there is a degree of consensus about how to capture the phenomenology of action in terms of SoA. According to the basic definition provided by Gallagher, SoA is the sense that I am the one who is causing or generating an action, and comprises multiple aspects ranging from first-order experience linked to intentional aspects and bodily movements to second-order reflective attribution (Gallagher, 2007). Further conceptual refinement led to the distinction of a feeling of agency and a judgment of agency as different levels of SoA (Synofzik et al., 2008) and the framework of optimal cue integration building on prediction and postdiction (Synofzik et al., 2013)^7^. Based on the phenomenal states associated with agency, experience of a movement can take different shapes, such as an action (of one's own), an action that one is of control of, an action that one is performing with a certain degree of effort, or an action that one is performing freely (Bayne, 2008).

All these approaches share the common ground that SoA is a *phenomenal echo* of sub-personal motor processes, i.e., “Awareness is a delayed and attenuated version of motor performance.” (Haggard and Johnson, 2003, p. 81). Even if Haggard and Johnson stress the aspect that the phenomenology of action becomes more accessible during tasks which ask for active engagement of the agent, such as rehabilitation or motor learning and recreational activity, their far-reaching observation is not further elaborated. Common approaches to SoA and the phenomenology of action in general still take an *experiential stance toward agency* (Horgan et al., 2003, Bayne, 2008) which binds any phenomenally present agency to the dimension of perceptual experience. This experiential stance toward agency depends on the common triadic structure of (1) a subject of experience, i.e., the agent, (2) the experiential content, i.e., the phenomenal state of an action, which depends on (3) the object of experience, i.e., (aspects of) the physical movement. As will be shown by means of analyzing the functional organization of SoA, this experiential stance and the dependency of the state (2) on the object (3) inevitably renders agency a *post-hoc* phenomenon of sub-personal motor processes so that agentive experience in terms of SoA falls under an experiential caveat and is not supposed to play any efficacious role (Bayne and Levy, 2009).

### 3.2. Binding: making subjective agency impossible

In the following, we will analyze the *functional organization of SoA* within Pacherie's model (cf. Table 1). Beginning with a brief presentation of Pacherie's framework (Section 3.2.1), we will then focus on the organizational principle, the implementation of behavioral functions and the resulting type of consciousness (Section 3.2.2; cf. Table 1). This analysis will clarify why efficacious action consciousness is generally made impossible by unifunctional integration.

**Table 1:** **Functional organization of unifunctional (SoA) and multifunctional (SA) integration**.

**Functional organization**	**Organizational principle**	**Implementation of behavior**	**Type of consciousness**
Unifunctional integration	Hierarchy: binding	Neuronal modules	SoA: experiential, non-efficacious
Multifunctional integration	Heterarchy: asymmetric reciprocity	Neuronal operators	SA: performative, efficacious

#### 3.2.1. Pacherie's approach to the sense of agency

Despite the fact that SA forms a cornerstone of folk psychology (Malle, 2004) and the organization of societal life in general, Libet set off the latest avalanche which seeks to explain any action consciousness as an epiphenomenal consequence of locomotor processing (Libet et al., 1983). Following corresponding accounts of the brain, action consciousness is captured by experiential (*post-hoc*) SoA and epiphenomenally attached to sub-personal processes in the brain (Flohr, 1991, Metzinger, 2003).

In light of this development, Pacherie's approach is insofar of interest as she generally argues for *conscious* agency and considers processes of action initiation and control that not only represent goals or executed actions, but more actively organize and structure motor processes (Pacherie, 2014). This position possibly leads a way to SA. Yet on the other hand, she proposes a complex model which attributes SoA to a cybernetic model of action specification (Pacherie, 2008, also Kumar and Srinivasan, 2012 drawing on Clark, 2013). It is exactly this latter model which renders conscious agency, as Pacherie proposes, impossible. Regarding the aetiology of agentive experiences, Pacherie goes for a comparator-based approach according to which bodily action is initiated and controlled by inverse and forward models in a central monitoring framework (Frith et al., 2000) which basically follows a cybernetic setup of control mechanisms (Wolpert, 1997).

The comparator model serves to instantiate a dynamical model of intentions consisting of three hierarchical levels from distal D-intentions down to proximal P-intentions and motoric M-intentions (Pacherie, 2008). *D-intentions* consist of beliefs and desires and therefore concern the overall decision-making and rational control of bodily actions. *P-intentions* form a link between the rational level of D-intentions and motoric implementation. They integrate D-intentions and the situational constraints of a particular action (situational anchoring) and control the execution of an action. Finally, *M-intentions* concern the selection of motor-programs and serve for basic motor control. Based on this model, Pacherie breaks SoA down into the *sense of intentional causation*, the *sense of initiation* and the *sense of control*. These phenomenal agentive experiences of SoA are explained by attributing them to the neural processes underlying D-, P- and M-intentions.

#### 3.2.2. Hierarchical binding and phenomenal counterparts

The standard account of unimodal integration (binding) builds on temporal synchrony. According to von der Malsburg and his correlation theory (von der Malsburg, 1994), neurons which process input of the same sensory object, are supposed to fire in temporal synchrony^8^. By means of roughly simultaneous fire rates, (populations of) neurons which relate to one and the same stimulus, synchronize even if the neurons are located in rather distant areas of the brain. Every external object can therefore evoke a certain representational pattern in the brain, a so-called assembly. These assemblies are supposed to bring about the homogeneous phenomenal consciousness of objects as single entities in that unimodal representations of an external object are bound together into one coherent neural state (Metzinger, 1995). In this view, conscious experience depends on the integration of basic sensory modalities and therefore emerges as an epiphenomenal higher-order process.

While the standard account of binding aims at binding features derived from sensory perception, Pacherie uses the underlying mechanism also for the integration of behavioral functions, referring to it as *efferent binding* (Pacherie, 2008). Based on the comparator model, a number of behavioral functions can be identified in her framework:^9^

- Comparator function
- Prediction of a movement
- Feedback of a movement
- Awareness of an intention to move
- Awareness of movement onset
- Motion initiation
- Motion supervision
- Motion execution

Pacherie explains the *sense of intentional causation* as the result of a comparison between the prediction and the feedback of a movement and the subsequent binding of movement and consequence. This type of efferent binding is also discussed as intentional binding (Haggard, 2005, Moore and Obhi, 2012). The *sense of initiation* results from binding the awareness of an intention to move and an awareness of movement onset. The *sense of control* depends on the comparison between desired, predicted, and actual states of a motion.

The behavioral functions underlying these senses which constitute SoA are mapped onto neural modules (modularization) so that there are specific brain areas, so-called neural correlates (of consciousness) (NCC) (Chalmers, 2000, Kühn et al., 2013), which instantiate behavioral functions. Thus, according to Pacherie's model SoA is the result of the integration of independent neural modules which implement the corresponding behavioral functions. One example is the supposed implementation of the comparator model by the posterior parietal cortex (PPC) which concerns the comparison of self-produced actions and their visual consequences, the cerebellum which concerns discrepancies between predicted and sensory consequences of actions and possibly the extrastriate body area (EBA) of the visual association cortex regarding visuo-motor incongruence (David et al., 2008). Accordingly, the sense of intentional causation is supposed to result from the bound (synchronized) activity of neural modules such as PPC, the cerebellum and EBA for comparison and modules for prediction and feedback processing which are possibly implemented by the supplementary motor area (SMA) (Eccles, 1982, Pfurtscheller et al., 2014). Resulting from the bound activities of neuronal modules at the basic level of processing, SoA emerges at higher levels of processing. The hierarchical binding of the behavioral functions constitutes SoA as “phenomenal counterpart[s]” (Pacherie, 2008, p. 193) which are epiphenomenally attached to cybernetic control mechanisms^10^.

Considering the hierarchy of unifunctional integration (with locomotory modules at the bottom and SoA at the top), it is the *temporal organization* which renders SoA inefficacious. As the neural modules work independently at the basic neuronal level, SoA follows on their independent activities. SoA occurs only *after* the proprioceptive loop has been closed as the comparator model depends on the efferent neural signal as well as on the afferent signal of consequential sensation of the intended motion. Accordingly, the *sense of intentional causation* is not efficacious as it relies on the afferent feedback of an actual motion^11^. The same limitation holds for the *sense of control* which also relies on actual states of a motion and therefore depends on the closed proprioceptive loop. The remaining *sense of initiation* does not rely on any afferent signal and therefore conveys the impression to be a suitable candidate for efficacious action consciousness. Yet, a patient can try to initiate motion even if no movement onset occurs so that also the sense of initiation presupposes an already initiated motion.

The temporal dependency on the closed proprioceptive loop and therefore on the integration of independent neural modules renders SoA a mere phenomenal counterpart of sub-personal motor processes. As a purely experiential consciousness, a phenomenal counterpart cannot play any efficacious role because it merely follows on locomotory events instead of effecting the latter. Moreover, SoA immediately vanishes once the corresponding locomotory mechanisms are out of order as in the case of patients with locomotive impairments. These findings show that *SA as efficacious action consciousness does not fall under common experiential action consciousness such as SoA*.

### 3.3. Subjective agency as a phenomenal performance

Regarding the results of robotic neurorehabilitation which gave rise to identify SA (Section 2) and the exclusion of SA from experiential action consciousness (Section 3.2), we suggest a preliminary working definition of SA as *phenomenal performance*. Accounts such as Chisholm (1966), O'Connor (2000) argue for something like SA on a conceptual level. But besides a certain conceptual plausibility, it is also important to fix the conscious phenomena of action initiation in an empirically verifiable manner^12^.

SA is consciousness of an action *during* its initiation and therefore occurs previous to visible motor behavior. On the one hand, SA, just as SoA, bears a certain qualitative state of consciousness and *phenomenal* content (Nagel, 1974). The agent brings to mind that she is about to move (e.g., to move to another place by forward walking). In healthy agents, the volition just passes by as the intended motion is immediately implemented. If the motion requires efforts (e.g., walking uphill), the volition is phenomenally stronger and includes exertion. And in case no bodily motion occurs, the volition might even be stronger in terms of futile attempts of initiation. On the other hand, SA is a prospect of the intended motion. The phenomenal content of SA is present in the very moment of initiation and not given after its initiation. In the moment of initiation, one acts voluntarily (e.g., starts to move to another place by forward walking) so that the conscious content of SA is equal to the voluntary initiation of that action and therefore comes as a “performance.” Taking together the phenomenal (qualitative) presence of SA and its performative content, we suggest the working definition of *phenomenal performance* in order to describe SA as a distinct type of efficacious action consciousness. In contrast, SoA is bound to intentional objects of experience (here aspects of motor behavior) and therefore relates to already executed acts. It can be characterized as a *phenomenal representation* of motor behavior.

In sum, the rehabilitation scenario yields particular evidence for SA in that the patient can make efforts to move consciously comparably with the conscious modification of thought or behavior during cognitive-behavioral therapy. Even if the patient's efforts to move do not result in any motion, SA still bears a phenomenally present performative act, and the corresponding neural signal occurs. Thus, even in the case of locomotory impairment, SA is still efficacious in releasing an efferent neural signal. But SA does not necessarily imply an awareness that one acts in terms of the action as an intentional object of experience as spelled out by SoA. Regarding the robotic rehabilitation scenario, SoA also plays an important role *after* motion has been initiated and implemented with the help of the robot. The patient receives different kinds of feedback, such as proprioceptive and visual feedback of her own motion. This information is also supposed to play an important role in the process of rehabilitation (Kawamoto et al., 2013). Thus, there are different types of experientially based consciousness of one's action, as SoA shows. But this phenomenal representation has to be distinguished from SA as a phenomenal performance.

## 4. Multifunctional integration and subjective agency

Hitherto, SA has been, firstly, identified as efficacious action consciousness (Section 2) which does, secondly, not fall under experiential action consciousness and comes as phenomenal performance (Section 3). As unifunctional integration or binding is not sufficient to explain SA's efficacy, the question arises as to what is needed in order to explain SA as efficacious and therefore immediate (instead of epiphenomenally attached) action consciousness. In the following, we will present a multifunctional approach to SA which could also be adapted for voluntary control of thought or combinations of thought and behavior as in cognitive-behavioral therapy. For this purpose, we will argue that the brain should be conceived in a way that allows the neural implementation of SA (Section 4.1). Then we suggest a functional organization of SA in terms of multifunctional integration (Section 4.2) and some general hypothesis on neurorehabilitation following SA (Section 4.3).

### 4.1. Not underestimating the brain

Whereas it should be the task of any scientific research about consciousness to explain what actually occurs in our conscious life, the current situation literally seems to have reversed. Instead of finding a conception of the brain which suffices for obvious phenomena such as SA, the latter are generally refuted by the prevailing conception of the brain as a representational device (cf. also Section 3.1). Hence, the situation arises that an obvious phenomenon such as SA is not allowed to be a conscious *and* efficacious phenomenon at the same time. This problem of recognizing SA stems from the underlying assumption of what the brain is capable of. If consciousness and cognition, as shown in Section 3.2, are supposed to result from neurocomputational brain processes, then the former can only achieve what the latter allow for. This bias excludes conscious processes from being efficacious regarding bodily action.

From a biological perspective Latash is making the same point when he explains that a biological system as the brain is explained in cybernetic terms which have originally been developed for much less complex systems such as the control of missiles (Latash, 2008, p. 323). In face of fundamental limitations of representational and information-theoretic explanations of consciousness (Eimer, 1990, Grüneberg, 2013), an analog point can be made here. Information-processing, which is mainly inspired by computational approaches and lies at the ground of neuroscientific approaches to cognition and behavior, does not capture complex intelligent behavior such as SA. Accordingly, from the viewpoint of SA, the fundamental questions arises why consciousness should *necessarily* and *exclusively* be experiential (*post-hoc*) and, subsequently, how to extend our understanding of the brain in order to include SA. As well as SA as multifunctional integration and in general the idea of multimodality, the concept of plasticity can be seen as another striking example that sticking to a certain conception of the brain avoids the recognition of its capabilities (Rubin, 2011). So it is important to continuously question the explanatory framework underlying the brain (Perruchet and Poulin-Charronnat, 2012).

### 4.2. Functional organization of subjective agency

Analogous to the functional analysis of SoA in Section 3.2, the functional organization of SA will be clarified in terms of the organizational principle, the implementation of behavioral functions (Section 4.2.1) and the resulting type of consciousness (Section 4.2.2; cf. Table 1).

#### 4.2.1. Heterarchy: asymmetric reciprocity

SA comprises the behavioral functions of voluntary initiation and the respective motor programs. Both can be distinguished as both can be performed independently of each other. While initiation can refer to other behavioral patterns such as cognitive behavior (Bayne and Montague, 2011), motor programs can also be initiated automatically without any contribution by the conscious agent. However, in case of SA both are integrated in a way that makes SA an *efficacious* action consciousness so that hierarchical binding with motor behavior at the basic level (as in case of SoA) is not any more feasible. Instead, we draw on the *heterarchic principle of asymmetric reciprocity* (Grüneberg, 2013, ch. 7, 8, Grüneberg and Suzuki, 2014) in order to explain the integration underlying SA. The general idea of asymmetric reciprocity is that action consciousness depends on a bidirectional relation of voluntary and automatic behavior with the former prevailing the latter. Such a bidirectional and asymmetric relation is what McCulloch (1945) and Günther (1971) call a heterarchy. A heterarchic relation allows for the *simultaneous* and therefore reciprocal activity of independent elements in a network so that behavioral functions are implemented reciprocally and at the same level of neuronal processing. At the same time, the heterarchic relation allows for one element governing other elements in that it includes a hierarchic and therefore *asymmetric* moment. In contrast to a strictly hierarchic setup where the governing element is predetermined by the hierarchy, the governing element in a heterarchy can change depending on the situation.

From this viewpoint, initiation as voluntary behavior and motor programs as automatic behavior asymmetrically depend on each other for the sake of SA. On the one hand, SA depends in two respects on motor programs. Firstly, if the agent wants to initiate a movement, the agent must be able to access her actuators. This job is done by motor programs (Ivanenko et al., 2004, Lacquaniti et al., 2012) which activate the locomotor system on a synergetic level (Latash et al., 2007). Voluntary behavior is enabled in that a voluntarily initiated motion is automatically executed after its initiation so that, for example, the agent can turn her attention to other tasks (Gallagher, 2006). Thus, automatic motion is not a contradiction to voluntary initiation, but the latter builds on automatic motor resources which comprise learned and habituated motor behavior and allow for new motor behavior. Secondly, if the agent selected a certain motor program, she is constrained to the respective motion and will move correspondingly. Even if she immediately modifies her motion by selecting a different motor program, every act of initiation is bound to its previous selection. Thus, any selection depends on the currently running motor program. Regarding the dependency of motor programs, a selection out of the pool of available motor programs is necessary in order to allow for coordinated (goal-directed) motion. Without a selection, no movement would occur. Thus, motor programs ask for a controlling instance. While this selection is often done by automatic selection, SA shows that this selection can also be done by the agent's voluntary initiation. According to this mutual dependency, initiation and motor programs are organized reciprocally^13^. At the same time, the selection of a specific motor program, i.e., the efficacy of initiation, implies an asymmetric relation in that initiation releases one specific motor program. In case of SA, the prevalence is in favor of the voluntary initiation with the motor program being selected so that initiation and motor programs are organized by *asymmetric reciprocity*.

Regarding SA as action consciousness, we suggest that its conscious appearance depends on asymmetric reciprocity. Generally, the content of phenomenal consciousness comprises particular objects. The main feature of phenomenal consciousness is the persistence and homogeneity of those objects—may these be physical objects externally perceived or cognitive contents such as thoughts, intentions or inner images. All these objects are characterized by the fundamental feature that they form homogeneous entities which can be distinguished from other entities and therefore identified as single entities (Metzinger, 2003). Analogous to the problem of experiential consciousness how objects composed of different features and mediated by different sensory modalities can appear phenomenally as homogeneous and therefore distinguishable objects, action consciousness faces the problem how the performing agent can distinguish between different behaviors so that these can become identifiable contents of phenomenal consciousness. Regarding SA, the question is how the agent can distinguish between her voluntary initiation and the initiated automatic motor program so that both become identifiable phenomenal contents.

We suggest that this can be done by means of asymmetric reciprocity. (It has to be noted that we are here in the first place concerned with asymmetric reciprocity as the basic organizational principle for the implementation of subjectivity (Grüneberg and Suzuki, 2014). Phenomenal consciousness (whether experiential or performative) as a particular instance of subjectivity asks for further relational processing which is figured out in more detail in Grüneberg, 2013, ch. 8). Take again the case of SA of forward gait. According to reciprocity, voluntary initiation and the motor program for forward gait mutually depend on each other and are implemented simultaneously so that they are contents of the same phenomenal state. At the same time, the voluntary behavior (that the agent seeks to walk forward) and the selection of the corresponding movement depends on the agent's self-determination (it is up to the agent how to behave). In turn, the content of the automatic behavior itself is pre-determined because a certain motor-program implies one particular motion (here forward gait). According to this asymmetry, both behaviors can be distinguished from each other in that the voluntary behavior (initiation) becomes distinguished *as voluntary* from automatic behavior (forward gait) *as automatic*. Voluntary initiation and the automatic motor program for forward gait can therefore be identified as particular phenomenal contents of one and the same state, i.e., SA of forward gait. It is this *mutual distinction* between voluntary and automatic behavior that distinguishes both behaviors from each other and allows for SA becoming conscious.

SA is also efficacious as the phenomenal content of SA is no other than voluntary initiation of a motor program. The conscious act does not refer to any higher-order or epiphenomenal level as in the case of SoA where the content of agentive consciousness (the phenomenal state) is different from the underlying behavioral functions (the object of that state) and therefore cannot bear any efficacy. For example while the sense of control, the object of experience, comprises the comparison between desired, predicted and actual states, it appears phenomenally as the feeling that one is in control of an action (Pacherie, 2008). In case of SA, the phenomenal performance can be directly identified with voluntary initiation of a motor program so that SA can be efficacious and conscious at the same time.

In the therapeutic scenario of neurorehabilitation (or cognitive-behavioral therapy), SA clearly prevails motor behavior. However, the same behavioral functions could also be arranged differently. Another scenario might include the ongoing walking motion while the agent is having a conversation. In this latter scenario, the motor behavior is not being prevailed by SA but performs automatically without being consciously initiated compared to the rehabilitation scenario. The automatic execution allows an agent to focus on other tasks such as motion related aspects (e.g., navigation) or tasks completely distinct from motion (e.g., conversation or observation of the environment during walking). Therefore, if motor behavior is not initiated voluntarily but performs automatically or is not performed by the agent at all, this behavior is not conscious as there is no mutual distinction with any voluntary behavior. It depends on a particular situation which kind of functional behaviors are implemented reciprocally so that a phenomenal performance such as SA might arise.

#### 4.2.2. Multifunctional integration: operators sharing functions

According to modularization, behavioral functions are implemented by independent neural modules so that the integration of several functions follows *after* each independent function has been activated. Therefore, unifunctional integration depending on binding comes as a *secondary integration*. In contrast, SA asks for a *primary integration* of behavioral functions, i.e., the behavioral functions have to be immediately activated as integrated functions. Such a heterarchy cannot be facilitated by unimodal (secondary) integration. For this reason, we argue that SA requires *multifunctional integration*.

In the following, we refer to the concept of the *operator* in order to neurally implement SA as multifunctional behavior. This means that both voluntary initiation and the motor programs have to be implemented at the same basic neuronal level. After identifying what Bassin et al. (based on the works of Bernstein) called “neuronal polysensority” (Latash et al., 2000, p. 136^14^), they proposed the concept of an *operator* in order to describe the modular (basic functional) units of the brain. Derived from control theory, an operator designates the particular design of a neuronal net which fulfills a specific operation in the neurodynamic processes of a brain region (Isomura et al., 2009). These operators can implement different behavioral functions and therefore come as the independent units of neural processing. For example, there are operators (neural circuits) that perform mathematical or action-related operations which can be shared by different functions (Latash, 2008) such as action planning, action initiation or learning. It is beyond the scope of this paper to identify particular neuronal operators. But, regarding SA, it can be suggested that there should be operators for the decision for, selection and release of a motor program which implement voluntary initiation. Neural circuits in the SMA and the insula might be possible candidates for implementing these operators (Eccles, 1982, Pfurtscheller et al., 2014). Other operators would comprise synergistic components which implement motor programs (Latash, 2008)^15^. In contrast to unimodal integration, multifunctional integration implies that behavioral functions are not directly (one-to-one) implemented by neural modules so that each single function has to be activated independently and then integrated. Instead, multifunctional operators implement behavioral functions *simultaneously as integrated functions* in that single functions are only realized reciprocally and in the context of a comprehensive multifunctional behavior such as SA. Due to their multimodal/-functional operationality, operators allow for a primary and therefore multiple integration of behavioral functions.

Multifunctionality also implies that SA is a non-localizable function. There is no rigid modularization on the neural level according to which SA could be attributed to a NCC. Building on operators, there are not only several brain areas involved in SA but also the spinal cord^16^ so that SA as a behavioral function is attributed to the entire agent as an embodied and conscious entity.

In sum, the functional organization of SA as a multifunctional setup resolves shortcomings of unifunctional integration of action consciousness. As SoA merely covers *post-hoc* experience and therefore neglects the efficacious nature of SA, the organization of the brain should be modified to that extent that phenomenal performance as an efficacious capacity can be implemented. For this purpose, we suggest the heterarchic relation of asymmetric reciprocity as the organizational principle and neural operators as the implementation of the functional organization of SA.

### 4.3. Improving neurorehabilitation by utilizing subjective agency

Currently, discussions on neurorehabilitation center around whether an active or passive approach is more effective (Belda-Lois et al., 2011). This issue concerns the degree to which a patient's active participation is required in order to activate and control the therapeutic device (cf. Section 2). A related issue concerns neurorehabilitation as a form of motor learning (Huang and Krakauer, 2009, Kitago and Krakauer, 2013). Whereas motor learning approaches also consider the effect of active participation in terms of initiation of a movement by the patient, they mainly focus on the ongoing execution of a movement and the subsequent learning effects.

From the viewpoint of SA, an active approach which stresses the importance of voluntary *initiation* compared to the execution of a movement is advocated. This leads to the following hypothesis: *(1.) Effects of neurorehabilitation are significantly increased by voluntary initiation which (2.) enables motor learning*. Regarding the neuronal dynamics, SA initiates the proprioceptive loop so that the patient executes motor programs successfully (cf. Section 2). This effect builds on the multifunctional integration of SA according to which voluntary initiation directly activates motor programs. Accordingly, a patient can initiate movement comparable to a healthy condition (Section 4) so that an active approach to neurorehabilitation is supposed to be more effective than a passive approach because the active rehabilitation entails activation of the entire processes related to the intended movement whereas the passive rehabilitation incorporates solely local processes that are directly related to the treated joints. Furthermore, utilizing SA in supervised and unsupervised learning scenarios with robotic devices, a patient will receive proprioceptive feedback regardless whether the trained movement was successful or asks for further improvement. This allows a patient to enter into a learning process even if execution of movements is limited. Thus, SA also comprises enabling conditions for motor learning so that voluntary initiation should be emphasized compared to motor learning which performs often automatically once a motion has been initiated. Both parts of the hypothesis can be tested within the robotic framework presented in Section 5 as there is also behavioral evidence for the efficacy of neurorehabilitation initiated by SA (Section 5.2).

## 5. Experimental evidence for subjective agency

### 5.1. Robotic case study: exoskeleton robot HAL

**Figure 2:**
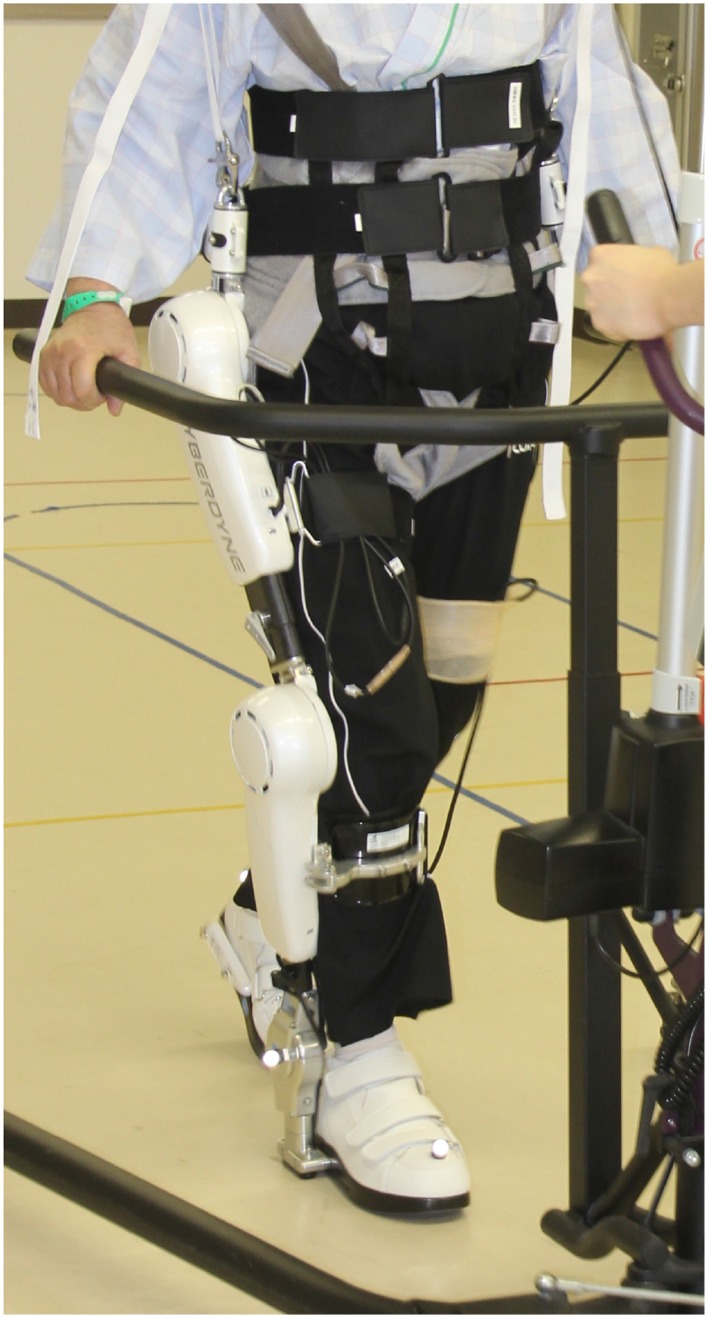
**Patient wearing HAL in a walking device (front view)**.

**Figure 3:**
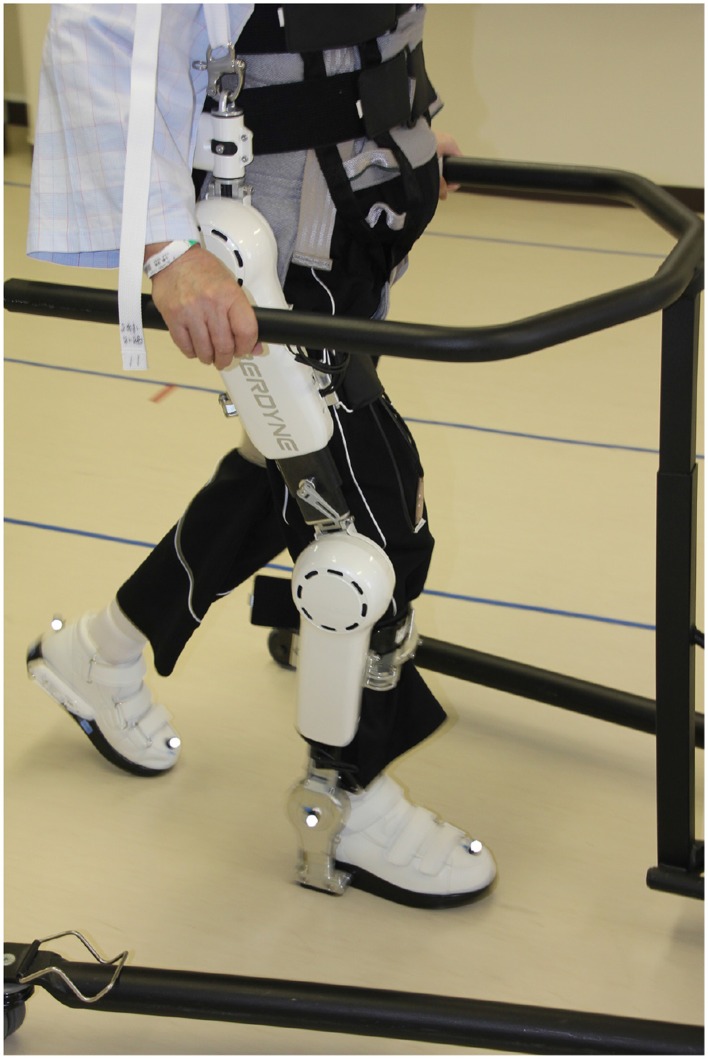
**Patient wearing HAL in a walking device (side view)**.

For the purpose of illustrating SA, we will present the exoskeleton robot HAL (hybrid assistive limb) (Sankai, 2006, Sankai, 2011) which is used for gait rehabilitation of spinal cord injury and stroke patients who suffer from severe impairments of motion (cf. Figures 2, 3). Currently HAL supports straightforward walking, standing up and sitting down. As different clinical studies show, HAL has successfully supported rehabilitation of 16 stroke patients (Kawamoto et al., 2013), 32 patients with stroke, SCI, muscoskeletal and other diseases (Kubota et al., 2013), and one patient with ossification of the posterior longitudinal ligament (OPLL) (Sakakima et al., 2013). Compared to mechanically based exoskeleton robots which facilitate passive support^17^, HAL makes use of biosignals and facilitates active support.

Drawing on the proprioceptive loop (cf. Figure 1 and Section 2.2), HAL's functionality can be described as follows: After the patient has been equipped with HAL, she voluntarily initiates a motor program for forward walking. HAL's crucial feature consists of EMG sensors attached to the flexor and extensor muscles of hip and knee. By means of this sensors, HAL detects the efferent active neural signal released by SA. In case there remains enough neural activity in the leg muscles, HAL interprets the neural impulse from the brain as a command to support walking motion and generates torque so that leg movement is facilitated. An afferent signal of consequential sensation is reported back to the brain and closes the proprioceptive loop and thereby supports neurorehabilitation. Thus, the patient initiates HAL's online gait support so that HAL is able to close the proprioceptive loop by estimating the patient's intention to move (Suzuki et al., 2007). Without HAL these patients are not able to initiate the physical gait motion *efficaciously*. The motor program is indeed issued, but not actually implemented. The fact that with HAL they are able to move implies that patients are able to initiate sub-personal motor-processes consciously by means of their SA.

In sum, the HAL scenario illustrates how SA is implemented as multifunctional behavior depending on asymmetric reciprocity. Voluntary initiation is directly bound to motor programs for forward gait in that the patient seeks to walk forward. Reversely, motor programs for forward gait are only initiated due to the agents conscious efforts to walk forward. Thus, in that both behavioral functions are activated simultaneously with voluntary initiation governing the selection of motor programs, SA is multifunctionally integrated and comes as efficacious action consciousness.

### 5.2. Testable hypotheses building on subjective agency

There are two possible areas where SA leads to testable hypotheses. One concerns neurorehabilitation by means of robotic devices. For the purpose of robotic neurorehabilitation, two different approaches are pursued as described in Section 2. On the one hand, patients use robots which build on the physiological signals of the patients motion. As these signals directly represent the intended motion, patients with locomotor impairments are enabled to initiate motion voluntarily (by themselves) while using a robot device. On the other hand, exercise is done by passive motion in that a therapist or a robot moves the patients limbs or body irrespective of motion initiation by the patient. In case that the human locomotor system would not allow for SA but only for SoA, therapeutic outcome of these two kinds of therapy would make no significant difference.

There are some reports on the importance of participants efforts to initiate motion (Hogan et al., 2006; Eitam et al., 2013) during motor learning (Lotze, 2003) or hand rehabilitation (Takahashi et al., 2008) as well as the examples of the lower-limb exoskeleton robot that we discussed in the previous sections. Future analysis of the outcome of robotic rehabilitation could investigate the differences between the two approaches in a more evidence based manner. *A testable hypothesis concerns the extent of rehabilitative effects*. In case of SA, reflecting its characteristics as whole body phenomenal performance, whole body coordination including stability, efficiency in multiple muscle coordination, limb synergies and head/posture control during locomotion is improved while in the case of SoA only limb joint motion might be improved. This difference can be physically evaluated by means of motion measurement and analysis technology using 3D motion tracker and EMG sensors in addition to the conventional 10m walking speed test and by applying gait analysis methods which are commonly used in the field of behavioral science.

The other area concerns conscious initiation of motion and online control. Based on the functional organization of SA, experiments should focus on the link between voluntary initiation and motor programs as SA plays a major role in the selection of a single motion out of a pool of available motions. Of particular interest is the question how phenomenal performance controls motor programs, i.e., how an agent can shape her motor behavior by means of initiation and online control. In case of athletes, motion in competitive contexts entails a variety of extraordinarily rapid movements so that feed-forward control of motion is widely exploited whereas feedback control might be too slow to be included. Here, it should be considered to test conscious self-recognition of motion. In case of SoA, self-recognition reflects the conducted motion since SoA depends on the perception of represented motion. In case of SA, self-recognition might be rather different from the actually conducted motion. Considering that an athlete by means of SA might have learned an appropriate way of tricking sub-personal locomotor processes through training, she might in some situations be able to manipulate sub-personal processes much more effectively for better performance than by sending naive straight forward commands. *Thus, the subsequent hypothesis states that there are subjective motoric behaviors which allow for a goal-directed manipulation of motion*.

To test this hypothesis, motion measurement technology can be used again. First athletes are interviewed how they control motion and what is the key variable to control for example the height of a jump and the angle of rotation during turning in their specialized sports motion. Then we can compare their self-recognition of the motion to the physically measured motion. Differences between these two measurements can support the existence and efficacy of SA. Predictions include that SA concerns the global synergetic level of motion and rather not kinematic and kinetic details of motion. Moreover, the conscious access to or initiation of motion is supposed to contain highly subjective motoric behaviors which are not necessarily observed in objective kinetic and kinematic measurements.

## 6. Conclusion

Robotic rehabilitation yields evidence that there is action consciousness prior to conducted motion. A similar finding can also be derived from cognitive-behavioral therapy where the voluntary involvement of the patient does also form an essential part of the therapeutic process. Based on this evidence, we argued for SA in terms of voluntary initiation of motor programs for movement. By analyzing robotic neurorehabilitation and introducing the proprioceptive loop, it could be concluded that, firstly, SA as an efficacious conscious act does exist. Secondly, we distinguished SA from common action consciousness by means of an analysis of the functional organization of SoA that showed that SoA depends on unifunctional binding which inevitably leads to *post-hoc* and therefore inefficacious action consciousness. Because SoA is implemented by independent neural modules corresponding to the behavioral functions, consciousness emerges not until the functions are integrated (bound) and therefore beyond functional efficacy. Therefore, SA implies a different type of action consciousness and has been identified as a phenomenal performance: a conscious act which consists of voluntarily initiating motor behavior.

For the sake of implementing SA, we suggested multifunctional integration of the behavioral functions underlying SA. Drawing on the heterarchic principle of asymmetric reciprocity, voluntary initiation and motor programs can be integrated at the same neuronal level simultaneously with the prevalence of initiation. We argued that it is the *mutual distinction* between voluntary and automatic behavior that allows for SA becoming conscious. Regarding the neural implementation of SA, we referred to the concept of the multifunctional operator which forms the basic neuronal module and is shared by different functions so that the activation of behavioral functions goes hand in hand with their integration. This means that the behavioral functions are not implemented independently as modules and then possibly integrated, but immediately integrated at the time of their activation. The multifunctional integration makes SA conscious with functional efficacy. Finally, we presented a robotic case study as experimental evidence for SA and sketched experimental setups of neurorehabilitation and athletic motion control in order to gain behavioral evidence for SA.

In sum, we propose that there is the phenomenal performance of SA as a type of efficacious action consciousness. Our analysis showed that an unifunctional approach to the brain is too narrow in order to capture the complexity of human behavior. Future research should seek to integrate multimodal input and multifunctional behavior. For this purpose, research in bodily motion forms an instructive starting point as movement implies a broad range of sensory and behavioral processing which are inherently integrated.

### Conflict of interest statement

The authors declare that the research was conducted in the absence of any commercial or financial relationships that could be construed as a potential conflict of interest.

## Footnotes

^1^If not otherwise specified, we will subsume the different senses related to action (sense of agency, ownership, intention, control etc.) to the sense of agency (SoA).

^2^Legrand also uses the concept of “subjective agency” for the pre-reflective condition of phenomenal experience (Legrand, 2007) and thereby remains within the common understanding of agentive as experiential consciousness. In turn, we relate SA to the agent's capacity to initiate action voluntarily and consciously.

^3^Numerous examples for upper limb devices depending on biosignals or mechanical control can be found in Maciejasz et al. (2014); for an example of a lower limb device building on biosignal processing see Section 5.1.

^4^For current purposes, the fact is crucial that the patient's ability to move is (partially) restored whereas it exceeds the scope of this paper to specify which brain regions in particular are affected. For further considerations of the neural implementation of SA cf. Section 4.2.

^5^The patient's capacity of SA can be generalized to healthy subjects as healthy subjects are also capable of a particularly active engagement in physical motion which exceeds automatic motion (Haggard and Johnson, 2003).

^6^Here we skip the question whether voluntary initiation refers to one motor program or possibly a complex of motor programs in order to initiate bodily motion.

^7^See David et al. (2008) for an overview of different accounts on SoA, distinguishing between the comparator model, simulation theory, intentional binding, and the multifactorial two-step account. Regarding gait, Kannape and Blanke argue that the central monitoring framework (comparator model) suffices for gait agency despite certain necessary refinements in case of gait as a whole body motion (Kannape and Blanke, 2012).

^8^While temporal synchrony can be regarded as the standard account, also other accounts have been proposed; cf. Cleeremans (2003).

^9^For the sake of simplicity and as the general mechanism of binding remains the same, we do not further distinguish between non-sensory behavioral functions which control motion or which entail sensory input related to motion such as proprioceptive feedback. For the same reason, we also ignore the distinction between awareness and experience of action.

^10^The explanation of SoA in terms of phenomenal counterparts of cybernetic processes could also be extended to the general explanatory conflict that the explanandum (SoA) becomes superfluous in face of the explanans as cybernetic processes do not necessarily imply any phenomenal experience of agency, cf. Grüneberg (2013), chapter 5.

^11^Research on processes of action selection suggests that there is a prospective generation of SoA which does not rely on afferent signals (feedback) (Chambon et al., 2014). On the one hand, action selection should be regarded as preceding SA. However, the authors suggest that this prospective generation merges fully in the *post-hoc* experience of SoA, so that it is not obvious how the prospective generation should be efficacious.

^12^The proposed account of SA might at a first glance be similar to O'Connors' concept of agent causation (O'Connor, 2000) as in both cases the agent is supposed to be the cause of her action. The most important difference is that the phenomenality of SA refers to the real (embodied) agent and does not imply any metaphysical foundation in terms of O'Connors' agent.

^13^Analogously, Chalmers et al. argue that so-called higher-order (conceptual) and lower-order (perceptual) processes necessarily depend on each other and thereby show that alleged high-level or subjective cognition is already at play in so-called low-level cognition (Chalmers et al., 1992). The same counts here in that locomotor processes involve subjective selection processes.

^14^The cited paper is a translated reprint (Latash et al., 1999, Latash et al., 2000) of Bassin et al. (1966) which was originally published in Russian language.

^15^Downward causation might serve as a comprehensive framework of the neural implementation of SA (Murphy et al., 2009).

^16^Control of movement roughly involves the spinal cord and brainstem circuits (lower motor neurons), the motor cortex and brainstem centers (upper motor neurons), the cerebellum and the basal ganglia (Purves et al., 2011). Depending on lower motor neurons and the generation of synergies in the spinal cord by central pattern generators (Grillner and Wallen, 1985), the neural control of movement encompasses the entire central nervous system.

^17^Examples include Lokomat (Colombo et al., 2000), ALEX (Banala et al., 2009) or AutoAmbulator (Fisher et al., 2011).
